# Robotic Semi-Automated Transcranial Doppler Assessment of Cerebrovascular Autoregulation in Post-Concussion Syndrome: Methodological Considerations

**DOI:** 10.1089/neur.2020.0021

**Published:** 2020-11-25

**Authors:** Danyal Z. Khan, Michal M. Placek, Peter Smielewski, Karol P. Budohoski, Fahim Anwar, Peter J.A. Hutchinson, Manohar Bance, Marek Czosnyka, Adel Helmy

**Affiliations:** ^1^ Division of Neurosurgery, University of Cambridge and Cambridge University Hospitals NHS Foundation Trust, Cambridge, United Kingdom.; ^2^ Brain Physics Laboratory, Department of Clinical Neurosciences, University of Cambridge, Cambridge, United Kingdom.; ^3^ Department of Biomedical Engineering, Faculty of Fundamental Problems of Technology, Wroclaw University of Science and Technology, Wroclaw, Poland.; ^4^ Department of Neurorehabilitation, Cambridge University Hospitals NHS Foundation Trust, Cambridge, United Kingdom.; ^5^ Department of ENT, University of Cambridge and Cambridge University Hospitals NHS Foundation Trust, Cambridge, United Kingdom.; ^6^ Institute of Electronic Systems, Warsaw University of Technology, Warsaw, Poland.

**Keywords:** brain concussion, cerebral blood flow, cerebrovascular circulation, post-concussion syndrome, traumatic brain injury

## Abstract

Post-concussion syndrome (PCS) refers to a constellation of physical, cognitive, and emotional symptoms after traumatic brain injury (TBI). Despite its incidence and impact, the underlying mechanisms of PCS are unclear. We hypothesized that impaired cerebral autoregulation (CA) is a contributor. In this article, we present our protocol for non-invasively assessing CA in patients with TBI and PCS in a real-world clinical setting. A prospective, observational study was integrated into outpatient clinics at a tertiary neurosurgical center. Data points included: demographics, symptom profile (Post-Concussion Symptom Scale [PCSS]) and neuropsychological assessment (Cambridge Neuropsychological Test Automated-Battery [CANTAB]). Cerebrovascular metrics (nMxa co-efficient and the transient hyperaemic-response ratio [THRR]) were collected using transcranial Doppler (TCD), finger plethysmography, and bespoke software (ICM+). Twelve participants were initially recruited but 2 were excluded after unsuccessful insonation of the middle cerebral artery (MCA); 10 participants (5 patients with TBI, 5 healthy controls) were included in the analysis (median age 26.5 years, male to female ratio: 7:3). Median PCSS scores were 6/126 for the TBI patient sub-groups. Median CANTAB percentiles were 78 (healthy controls) and 25 (TBI). nMxa was calculated for 90% of included patients, whereas THRR was calculated for 50%. Median study time was 127.5 min and feedback (*n* = 6) highlighted the perceived acceptability of the study. This pilot study has demonstrated a reproducible assessment of PCS and CA metrics (non-invasively) in a real-world setting. This protocol is feasible and is acceptable to participants. By scaling this methodology, we hope to test whether CA changes are correlated with symptomatic PCS in patients post-TBI.

## Introduction

Traumatic brain injury (TBI) is a global challenge. An estimated 50 million cases of TBI occur yearly worldwide resulting in significant mortality, morbidity, and global economic expense ($US400 billion per year).^1,2^ Mild TBI (mTBI), commonly defined by a Glasgow Coma Scale (GCS) score between 13 and 15, represents 90% of TBI cases.^3^ Post-concussion syndrome (PCS) refers to a constellation of physical, cognitive, emotional, and psychiatric symptoms including headache, difficulty concentrating, memory problems, depression, and anxiety.^4^ PCS is most often described in the setting of TBI, most commonly after mTBI.^5^ Its prevalence post-TBI is between 11 and 82%,^6^ with a highly variable clinical course.^6^ Indeed, PCS contributes to a considerable amount of the economic burden (due to medical costs and loss of working time/performance) after TBI. Its morbidity is pervasive, negatively impacting patients' quality of life across multiple domains.^2,5,7^

Despite the incidence and impact of PCS after mTBI, it is a poorly characterized entity owing to heterogeneity in definitions, diagnostic criteria, and patient populations.^6^ Similarly, uncertainty and debate exist regarding the underlying mechanisms of PCS, particularly persistent variants of the syndrome.^5,8^ Additionally, there appears to be a degree of overlap between non-vestibular dizziness and PCS syndromes—with both describing non-specific dizziness, often exacerbated by anxiety and hyperventilation.^9^ Non-vestibular dizziness, also known as chronic subjective dizziness or persistent postural-perceptual dizziness (PPPD), may or may not be preceded by TBI or other vestibular pathology.^10^ Post-traumatic dizziness is disabling—being an independent adverse prognostic indicator post-TBI.^11^ It proves difficult to treat when compared with non-traumatic dizziness/vertigo—again, related to poor understanding of underlying mechanisms.^12^ There may be a cerebrovascular component to the pathogenesis of several vestibular disorders, with recent studies suggesting dynamic links between vestibular system activation and cerebral blood flow (CBF).^13^

We hypothesized that impaired cerebral autoregulation (CA) post-TBI contributes to these clinical syndromes. Cerebral autoregulation refers to the myogenic ability of the cerebral vasculature to maintain adequate CBF over a range of arterial blood pressures (ABPs).^14^ Usually, CA is able to maintain stable average CBF values between a mean ABP (MAP) range of 50–150 mm Hg.^14^ On the other hand, the ability to change CBF in response to arterial CO_2_ is termed cerebrovascular reactivity (CRx).^15^ Consequences of CA and CRx failure exist as a spectrum, ranging from the extremes of chronic ischemia to hyperperfusion, and can be globally, heterogeneously, or locally distributed.^16,17^ Disrupted CA and CRx have been observed in all degrees of TBI, even when values for CBF, cerebral perfusion pressure, ABP, and intracranial pressure (ICP) are normal.^18,19^

Two complimentary (non-invasive) methodologies have provided evidence of the link between PCS and CA and CRx in the context of mTBI. Advanced imaging techniques such as arterial spin labeling (ASL) magnetic resonance imaging (MRI) have shown derangements in CBF in the context of mTBI and PCS symptoms.^17,20^ Similarly, blood oxygenation level-dependent functional MRI has shown changes in brain activation after concussion.^21,22^ However, these modalities are expensive, not widely available, lengthy, and not tolerated by some patients (e.g., those with claustrophobia). Some of these disadvantages are offset by using transcranial Doppler (TCD) as an alternative method. Using TCD studies has corroborated the hemodynamic disruption after concussion in real time—highlighting CRx and CA dysfunction in patients with mTBI with PCS symptoms.^15,23–26^ However, these studies are limited by small sample sizes, lack of longitudinal assessment, use of crude CA metrics, and frequent assessment of CRx without CA.

TCD provides a non-invasive and inexpensive method for repeated and robust assessment of CA.^27^ By calculating the correlation between changes in cerebral blood flow velocity (CBFV) at the middle cerebral artery (MCA) and changes in ABP (also measured non-invasively using finger plethysmography), a coefficient termed nMxa is derived. This has been found to correlate well (R = 0.755) with its invasive counterpart, Mx—a CA metric calculated using an arterial line and an ICP monitor to derive cerebral perfusion pressure.^28^ An Mx/nMxa value of >0.4 is indicative of cerebrovascular autoregulatory dysfunction.^28^ Further, Mx/nMxa correlates with other measures of autoregulation, (e.g., static cerebral autoregulation [sRoR] and CO_2_ reactivity)^29,30^ and may be less influenced by signal noise (as may be expected in alert patients with concussion) when compared with systolic-based autoregulation metrics. Additionally, the transient hyperaemic response ratio (THRR) represents another reproducible, non-invasive CA metric that correlates with TBI severity and TBI-specific outcome.^31,32^

We have adopted a fully non-invasive methodology to test the hypothesis that changes in CA are correlated with symptomatic PCS in patients with a history of TBI. We have assessed these metrics alongside PCS symptoms (and their impact on daily living) to assess the feasibility of this technique in a real-world clinical setting. In this article, we present our methodology and pilot experience as to its feasibility and acceptability for scaling.

## Methods

### Design

A prospective, observational design was adopted, and it was utilized at a tertiary university neurosurgical center (Cambridge University Hospitals).

### Ethical approval

The formal regional and institutional ethical board reviewed and approved this study (ethics reference number: 19/SC/0444). Written informed consent was ascertained from every human participant in the study. Patient data remains anonymized as a condition of ethical approval. Anonymized data are available upon request.

### Population

Inclusion criteria for patients were: having a history of TBI, with or without symptoms of PCS, presenting for follow-up at neurotrauma outpatient clinics. Exclusion criteria involved: age <16 years, history of pre-morbid symptoms overlapping with PCS, severe peripheral vascular disease (preventing finger plethysmograph use), and significant obstructive respiratory disease. A separate control group of healthy controls (asymptomatic patients without a TBI history) was also included.

Suitable patients were identified from routine neurotrauma outpatient clinic lists. Recruitment via informed consent was carried out by a research team member independent from the clinical team. For pragmatic and participant convenience purposes, recruitment and data collection were integrated alongside clinical assessments. Study participants had the choice of same-day study measurements (while waiting for their clinical appointments) or booking an alternative time.

### Data set

The measured data set covered multiple domains: demographics, symptoms, neuropsychological metrics, quality of life scores, and cerebrovascular and vestibular profiles. These domains were guided by the U.S. National Institutes of Health's (NIH's) National Institute of Neurological Disorders and Stroke (NINDS) Common Data Elements TBI with instruments within domains adapted to local expertise in instrument availability and delivery.^33^ Demographics included age, sex, medical/TBI history, cardiovascular medications, smoking history, and alcohol history (Supplementary Fig. S1). Symptom questionnaires included a 21-item Post-Concussion Symptom Scale^34^ (PCSS), a 9-point Vestibular Visual Analogue Scale (VVAS),^35^ and a bespoke vestibular symptom checklist aimed at characterizing dizziness episodes (if present; Supplementary Fig. S1).

The neuropsychological and cognitive assessment consisted of the Cambridge Neuropsychological Test Automated Battery (CANTAB): TBI battery.^36^ Quality of life and extended outcomes aiming to capture the wider context involved completion of the Glasgow Outcome Scale Extended (GOS-E)^37^ and the standard Short Form 36 Health Survey (SF-36).^38,39^ Cerebrovascular metrics were collected using an integrated TCD, finger plethysmograph, and bespoke software to calculate nMxa and THRR, as described below. The vestibular assessment involved assessing nystagmus at rest, a horizontal head impulse test, and a Fukuda stepping test (marked as normal, mildly abnormal, or strongly abnormal). The primary outcomes for the study were the calculation of nMxa, THRR, and PCS symptom severity. All episodes of data collection were timed, and the end-of-study feedback was collected from the participating patients and healthy controls. All measurements were taken by a clinician and researcher trained in the use of TCD, finger plethysmography, CANTAB, SF-36, PCSS, and vestibular assessment.

The inclusion of vestibular metrics is based on anecdotal experience of a subset of patients with non-vestibular dizziness and vertigo (with normal vestibular function tests and brain imaging) that appear to pose as a diagnostic and therapeutic corollary to PCS. We aim to investigate this group of patients as a distinct sub-group in the next phase of the study.

### Equipment and procedure

Demographic information was collected via paper form, and symptom questionnaires, the SF-36, and the GOS-E were completed via paper forms. CANTAB assessment was performed using an iPad Pro (second-generation 12.9-in, IOS 13.2.1, Apple, USA).

**FIG. 1.**
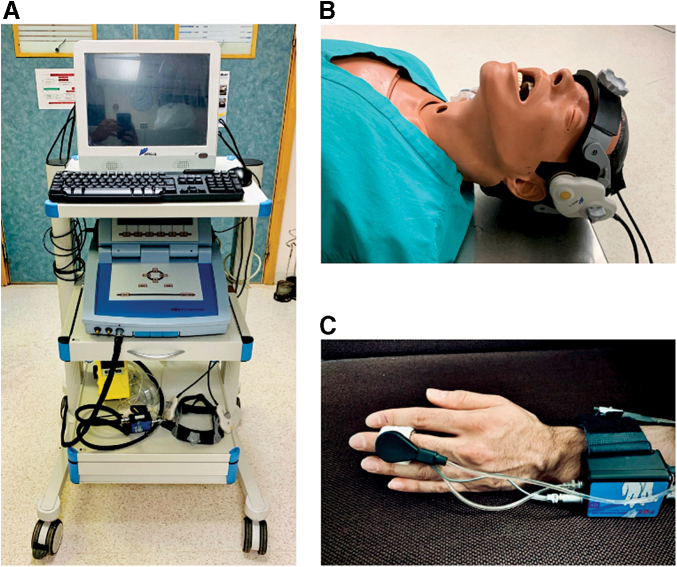
Setup and configuration. **(A)** Three-level trolley with Delica TCD system and Finapres system mounted. **(B)** Delica robotic probes in place, hosted within a bespoke headband. **(C)** Finapres finger plethysmograph *in situ*. TCD, transcranial Doppler.

Cerebrovascular profile equipment was mounted on a three-level cart: one level for the Delica computer system (Shenzhen Delica Medical Equipment Company, Shenzhen, China) with ICM+ software (Cambridge Enterprise Ltd., Cambridge, UK) installed, one level for the finger plethysmograph, and one level for storage of the robotic TCD headband and capnograph (Fig. 1A).

The Delica Robotic TCD headband was positioned so the Doppler probes (1.6 pulsed-wave, 81% amplitude, 1–2 MHz) were positioned over the transtemporal window (Fig. 1B). Field “scanning” is used to approximate to desirable coordinates and involves micro-adjustments of the robotic probe in a grid-like pattern. At each position on the movement grid, the system assesses signal presence and quality and produces a color-coded grid map to highlight probe positions where CBFV is best detectable.^40^ Anecdotally, the initial scanning function saves a considerable amount of time by identifying the general location of the MCA signal for probe placement. Similarly, the “search” function moves the probe in a spiral pattern, creating a spiral color-coded map to identify areas of strong signal.^40^

Once the probe is placed in this general area, a series of manual and robotic probe sensor adjustments are made to refine MCA flow velocity signal recording. Again, the scanning function guides this refinement phase. The MCA flow signal is found at a depth of 30–65 mm, with a flow velocity of 55 ± 12 cm/sec, continuous forward flow through systole and diastole, and a characteristic waveform.^41^ This is done via the Delica TCD software interface, which allows activation of robotic functions, changes in probe amplitude, gain, and depth; and visualization of the Doppler waveform. Once the signal is achieved, the probes are fastened in place. The Finapres finger plethysmograph (Finometer Pro, Finapres Medical Systems B.V., Enschede, the Netherlands) is then placed on the left middle finger (using the appropriately sized finger cuff), with wire positioning as per Figure 1B. The hand is kept still and at heart-level for the entire session.

TCD and Finapres recordings were gathered for 35–40 min (30 min for nMxa calculation, 5–10 min to account for artefacts). THRR was then calculated via the transient hyperaemic response test (THRT)—a series (two left and two right) of unilateral carotid artery compressions (5–7 sec). The tests were spaced 1 min apart to allow for autoregulatory system recovery between hyperaemic responses. Resting end-tidal CO_2_ (etCO_2_) was measured at the time of THRT using a handheld capnograph (Nellcor N-85 Microstream, Covidien/Medtronic Minimally Invasive Therapies, Watford, UK).

**FIG. 2.**
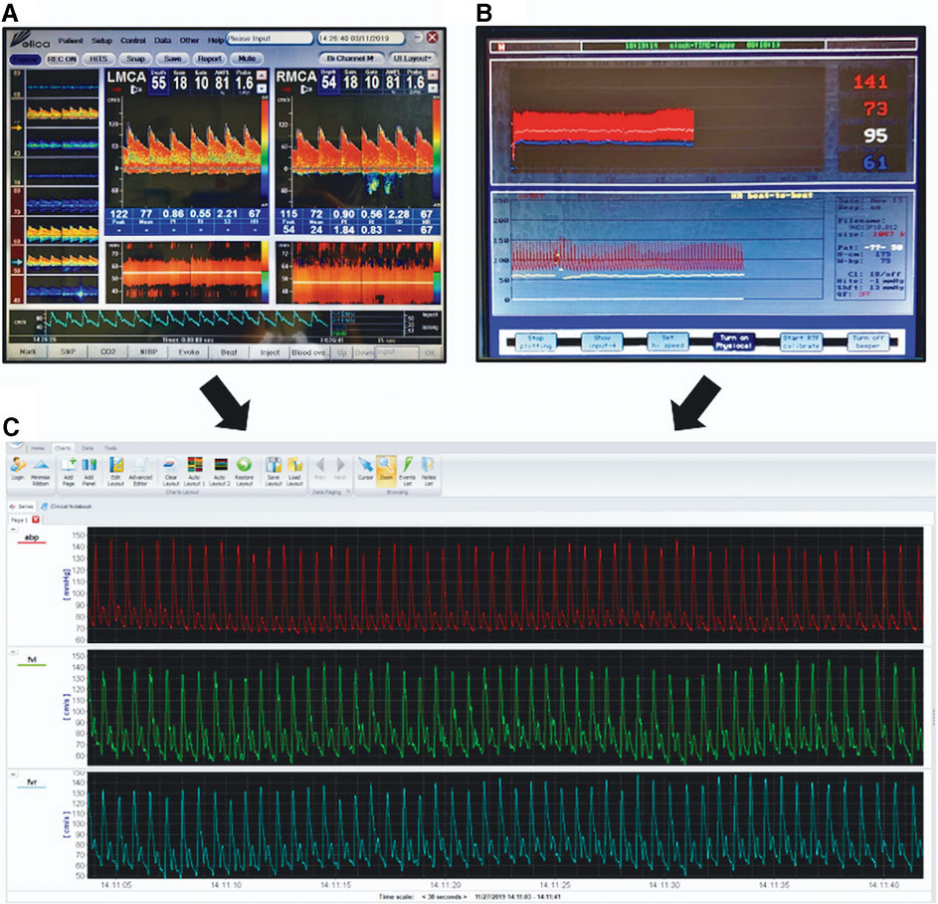
Real-time data collection. **(A)** Delica TCD interface displaying real-time waveform recording. **(B)** Finapres interface recording arterial blood pressure in real time. **(C)** TCD and Finapres data channeled into ICM+ software in real time. TCD, transcranial Doppler.

**FIG. 3.**
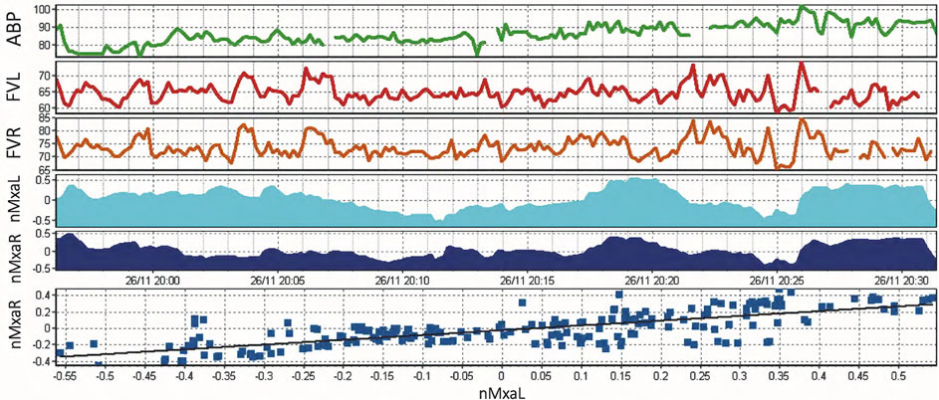
ICM+ interface with nMxa calculation. Artefact removal has taken place, representing missing chunks in data. The correlation coefficient of ABP (first row) and middle cerebral artery flow velocity on the right (FVR) and left (FVL) is calculated. This is represented by nMXaR and nMXaL, respectively. The last row displays a correlation line between nMXaR and nMXaL, in this case, R = 0.755. ABP, arterial blood pressure.

**FIG. 4.**
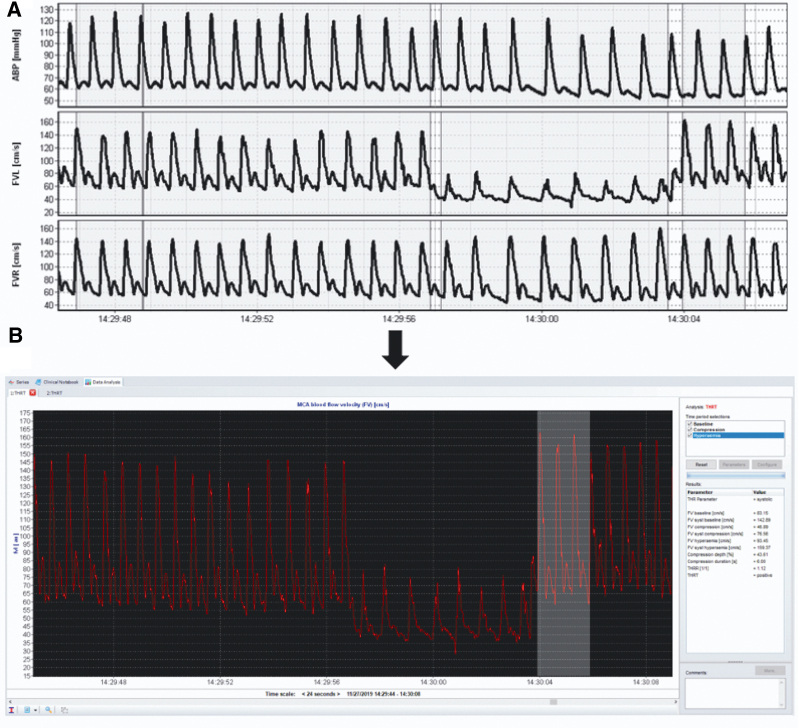
Transient Hyperaemic Response Ratio (THRR). **(A)** ICM+ raw data showing a decrease in FVL (flow velocity in the left MCA). **(B)** ICM+ data analysis tool is used to calculate THRR value. ABP, arterial blood pressure; FVR, flow velocity in the right MCA; MCA, middle cerebral artery.

Waveforms data from the TCD (Fig. 2A) and Finapres (Fig. 2B) were recorded using ICM+ installed on the Delica TCD (Fig. 2C) at a rate of 125 Hz. ICM+ is a bespoke clinical research software for physiological signal collection, analysis, interpretation, and presentation (https://icmplus.neurosurg.cam.ac.uk). After data refinement (artefact removal), nMxa and THRR were calculated by ICM+ as detailed below (Figs. 3 and 4). nMxa is computed fully automatically, whereas THRR calculations require marking of the relevant parts of the flow velocity waveform corresponding to different stages of THRT.

### Statistical analysis

Basic descriptive and summary statistics were generated for the demographic, symptom, quality of life, and neuropsychological data.

nMxa, calculated using ICM+, is the correlation coefficient between slow-wave (0.005–0.05 Hz) changes in ABP and slow-wave changes of CBFV^28^ calculated within a moving 5-min window. nMxa values have been recorded in healthy adults as 0.21 ± 0.16 (mean ± standard deviation [SD]),^42^ with a value of >0.4 thought to be indicative of cerebrovascular autoregulatory dysfunction in TBI.^28^ The THRR represents a complementary non-invasive CA metric (Fig. 3).^32^ It is the ratio of the MCA flow velocity change after brief ipsilateral carotid artery compression (for 59 sec causing >30% reduction in systolic flow velocity). On the restoration of flow, the first systolic peak is ignored and the next three systolic peaks are assessed and compared with resting flow velocities to calculate the THRR. A normal response, an indication of preserved CA, is a flow increase >9% (ratio ≥1.10) of baseline systolic velocity after compression.^32^

## Results

### Participant demographics

In total, 12 participants were initially recruited for this pilot study—6 healthy controls and 6 patients with TBI. Two participants were excluded from analysis after unsuccessful insonation of the MCA during TCD assessment. Among the 10 included participants, the median age was 24 years (range 22–63 years, *n* = 5) for the healthy controls and 30 years (range 20–48 years, *n* = 5) for the patients with TBI sub-groups (Table 1). The male to female ratio was 7:3. Relevant TBI and past medical histories of included participants are detailed in Table 1.

**Table 1.**
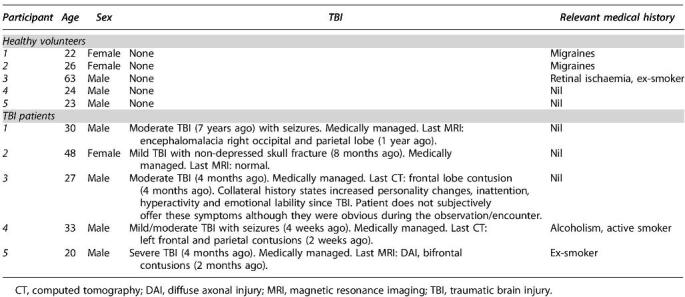
Summary Table Basic Demographics

### Symptom profiles

**FIG. 5.**
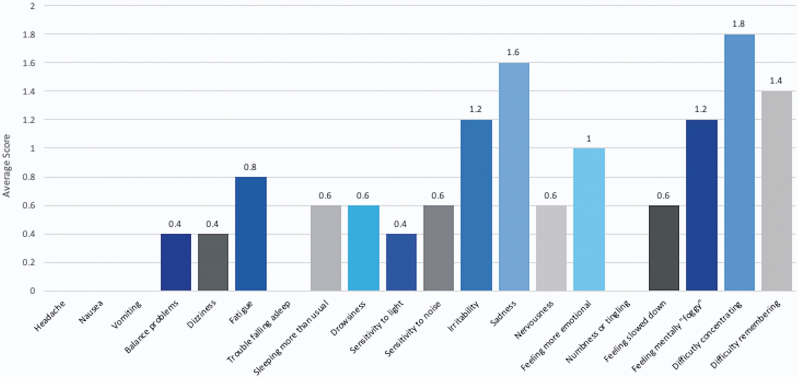
Post-Concussion Symptom Scale mean subcomponent scores for the TBI patient cohort. TBI, traumatic brain injury.

Median self-reported PCSS scores were 0/126 (range 0***–***2) for healthy participants and 6/126 (range 2***–***41) for the TBI patient sub-groups (Table 2) with a median duration of symptoms of 4 months (range 1***–***48). Aggregate scores for each of the 21 PCSS sub-components is presented in Figure 5. Only one participant (a patient with TBI) scored any points on the VVAS (score 16/90) and the vestibular symptom checklist (highlighting non-specific dizziness, lasting seconds to minutes, exacerbated by standing from sitting, stress, exertion, and complicated visual environments).

**Table 2.**
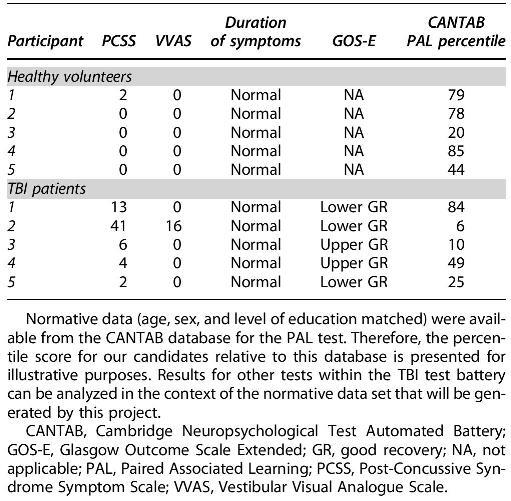
Summary of Symptom, GOS-E, and Neuropsychological Assessments

### Neuropsychological metrics and quality-of-life scores

The paired associated learning (PAL) sub-score of the CANTAB has robust normative data (age, sex, and level of education matched) available from the CANTAB database. Thus, the percentile score for our candidates relative to this database is presented in Table 2. Median PAL percentiles were 78 (range 20***–***85) for healthy controls and 25 (range 6***–***84) for the patients with TBI sub-groups. GOS-E scores for all of the patients with TBI groups fell within the Good Recovery (Upper or Lower) category (Table 2). Overall SF-36 scores (Table 3) were lower in patients with TBI (median 66.7%, range 64***–***78%) than in healthy controls (median 87.8%, range 87***–***90%).

**Table 3.**
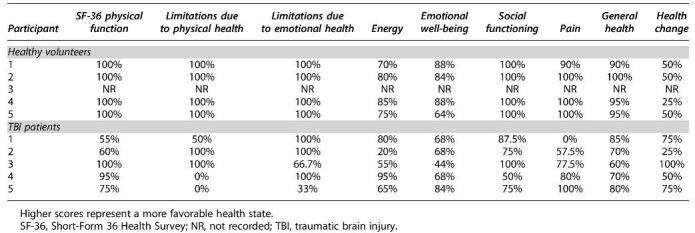
SF-36 Profile for Each of the Participants

### Vestibular and cerebrovascular profiles

Abnormalities in vestibular assessment (nystagmus at rest, horizontal head impulse test, and Fukuda stepping test) were not elicited in any of the participants (Table 4).

**Table 4.**
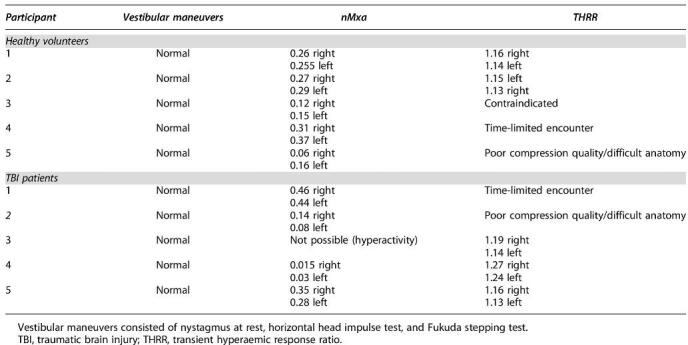
Summary of Vestibular and Cerebrovascular Profile Recorded per Participant

Two participants (*n* = 2/12, 16.7%) were excluded from the study as MCA signals were not clearly identified and therefore nMxa and THRR were not achievable, leaving five participants in each group for analysis. In one patient, MCA flow velocity signal was found and THRT was performed, but a reliable nMxa value was not obtainable due to patient hyperactivity (related to underlying PCS). nMxa was calculated for 100% (*n* = 5/5) of controls and 80% (*n* = 4/5) of patients with TBI with MCA recordings (Table 4). Median nMxa was 0.258 (range 0.06–0.37, *n* = 5) in healthy controls and 0.21 (range 0.02–0.46, *n* = 4) in patients with TBI. THRR was only calculated in 50% of participants, at normocapnic etCO_2_ levels. Interestingly, THRR values were more favourable in patients with TBI (median 1.15 [range 1.13–1.16, *n* = 2] in healthy controls, 1.18 [range 1.13–1.27, *n* = 3] in patients with TBI). The reasons for incomplete THRR data were as follows: 1) THRT was contraindicated due to previous retinal ischemic damage in one patient, 2) data recording was cut short owing to time limitations on two occasions, and 3) poor compression quality in two participants (related to difficult anatomy and a developing learning curve of the research team).

### Time taken and feedback

Median time taken for entire data set capture was 127.5 min (range 120–151). In terms of the general division of this time, setup of the TCD (including identifying and refining MCA flow velocity signal and initiating recording of Finapres ABP and TCD flow velocity with ICM+) generally took between 10 and 25 min—depending on the quality and ease of MCA signal attainment. nMxa measurement requires at least 30 min of data recording, therefore 35–40 min were taken to give room for potential signal artefacts. A set of THRTs followed, taking between 5 and 10 min. CANTAB assessment took approximately 35 min and the rest of the assessments (demographics, PCSS, VVAS, SF-36, GOS-E, and vestibular profile) generally took between 10 and 15 min.

Additionally, post-study acceptability was scored by participants from 1 to 5 (1: not acceptable, 2: somewhat acceptable, 3: acceptable, 4: very acceptable, 5: extremely acceptable). Six participants left feedback, all rating the study as at least acceptable (*n* = 2 for an acceptable score, *n* = 4 for very acceptable). Positives aspects of the study, such as “learning about non-invasive measures and cerebral blood flow” and its “interactive” nature, were highlighted by participants. In terms of the study challenges, the total “time-length” taken for measurements was raised, as well as “mild temporal pain” after prolonged temporal TCD probe application.

**FIG. 6.**
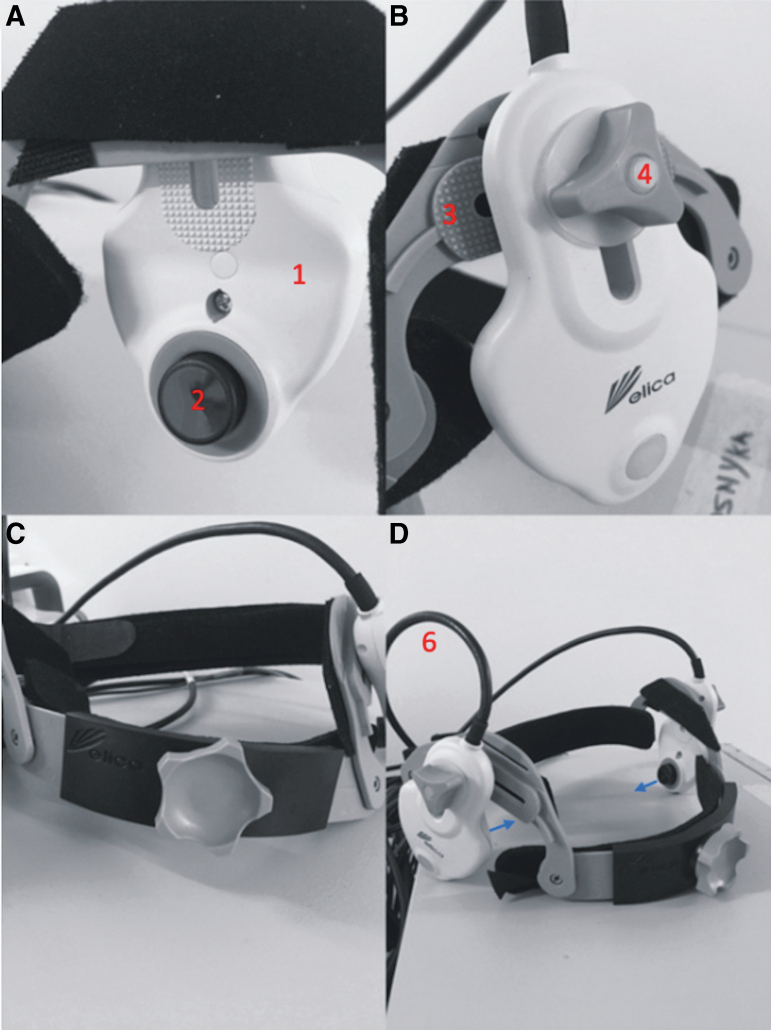
Robotic TCD headband constituents. **(A)** The probe arm is denoted by “1,” with the sensor at the *head* of the probe arm denoted by “2.” **(B)** The probe arm is mobile within the frame of the headband (“3”) and fastened in the desired position with a screw (“4”). **(C)** An anterior-posterior view of the construct with the probe arms on the left and right. **(D)** An oblique view of the construct, with the output wire (“6”) from the probe arm at its *tail*. This loops upwards and then backwards and may exert an anti-clockwise rotatory force on the probe arm. The blue arrows represent a tendency for the probe arms and sensor probes to apply pressure inwards toward the participant's temples when the securing screw is significantly tightened. Figure adapted with author permission from Zeiler and Smielewski.^40^ TCD, transcranial Doppler.

In regard to operator (D.Z.K.) feedback based on anecdotal experience, some technical points were noted. First, the Delica TCD probe arms (sensor probe at the head, wire at tail) are mobile within the headband construct and are secured into place by a screw system (Fig. 6). This screw is for fastening the base of the probe to the plastic headband frame. However, upon tightening, there is a degree of movement of the probe sensor (head of the probe arm) inwards toward the participant's head. Thus, at certain angles, the screws would have to be tightened to a maximal tightness to keep the probe arm in place for a prolonged period in an awake participant. This tightening can cause the sensor probe to press on the participant's temples, which, after some minutes of application, may cause pain at the temple or referred pain down the jaw. The close probe-skin contact may also displace the applied ultrasonic gel and interrupt MCA signal quality.

Similarly, the placement of the wire at the base of the probe arm means that when the probe's head (with the sensor probe) is angled inferiorly and the tail (with the probe wire) is therefore superiorly placed—gravity pulls the heavy wire down and displaces the probe sensor at the head. Ways to mitigate this include providing cushioning support to the wiring/probe trail (to offload the weight across the probe arm) or by further tightening the screws (but potentially causing pain for the participant). Further development of the screw mechanism and offloading of wire traction would provide a more stable and comfortable device for prolonged usage.

Finally, in terms of software challenges, the “track” function was difficult to use. This function is for following and maintaining the MCA signal by automatically adjusting the probe position and ultrasound wave properties in response to minor movements of the headband. However, we found that even when a high-quality MCA signal was found, when the tracking was engaged, any significant movement of the headband would trigger the system to adjust the probe/waves but focus on a random vessel signal that clearly was not the MCA. Other reports echo this difficulty with this robotic TCD system.^40^ Refinement of this function would be particularly useful for awake participants who will naturally move to a certain degree during the examination and may slightly dislodge the headband. We have been informed by the manufacturer that significant improvements have been made recently to address these problems, but we have not yet had a chance to verify those claims.

## Discussion

This pilot study has demonstrated a reproducible assessment of PCS symptoms and non-invasive measures of CA in five controls and five patients with TBI. The protocol has been applied in a real-world setting in an acceptable, feasible, and effective manner.

First, we developed a protocol and workstream with 100% (*n* = 6) perceived acceptability rates among both patients and healthy controls (via participant feedback)—although one of the patients was unable to tolerate staying still for the entire nMxa measurement. Study integration into ongoing neurotrauma clinics made recruitment of the target population feasible and convenient (for both the participants and researchers). Although our study requires a considerable time commitment (median 127.5 min), much of this time could fill periods patients would otherwise spend waiting at clinic appointments. We anticipate that with the refinement of our protocol, questionnaire assessments could be carried out on online platforms at the participant's convenience and the TCD monitoring period could be shortened.

Additionally, in developing this workstream, we have: established a dedicated CA trolley setup, licensed relevant software (ICM+, CANTAB), and developed local expertise in administering data instruments. This protocol captures a broad and multi-faceted data set in real time and therefore is readily available to treating clinicians. It is effective—in most participants, a combination of TCD and Finapres monitoring (for nMxa or THRR) was possible. In two participants (2/12, 16.7%) an MCA signal was not established bilaterally: one was an elderly female (age and female sex are associated temporal bone thickening) and the other was a young male who had a healed convexity skull fracture across the insonation site. This is in line with rates of temporal window failure, reported as generally between 5 and 20%.^43,44^ We anticipate that this would only be improved with further refinements in study technique and planning.

### Findings in the context of literature

The lack of a mechanistic understanding of PCS has made the identification of suitable biomarkers and specific treatments for PCS after mTBI difficult. Establishing a PCS biomarker or set of biomarkers will allow objective diagnosis, prognostication, and rigorous patient follow-up/recovery, and will facilitate methodological research.^45,46^ These biomarkers should ideally be non-invasive and easily assessed.

The benefits of nMxa and THRR lie in their non-invasive nature, with TCD central to both. Many groups have described the use of TCD to assess cerebral hemodynamics in patients with mTBI and PCS. Specifically, post-TBI CRx dysfunction (measured via assessing CBFV changes during CO_2_ challenges) has been found after mTBI in athletes and is associated with the presence of PCS symptoms, particularly headache and cognitive symptoms.^15,17^ Another non-invasive method employed in patients with mTBI is advanced MRI, which has reiterated the disruption of cerebral hemodynamics as a prominent and common feature in PCS patients. ASL has been used to find differences in CBF patterns in pediatric patients 40 days following mTBI.^17^ Patient without PCS symptoms showed a global decrease in CBF, as compared with healthy controls, whereas those with PCS exhibited a global increase in CBF.^17^ Longitudinal ASL MRI analysis of concussed athletes over 1 month has suggested decreased regional blood flow in the right superior temporal sulcus (transient) and right dorsal mid-insular cortex (persistent at 1 month) when compared with non-concussed athletes.^20^ However, MRI imaging is costly, time-consuming, and difficult to apply in a wider population (e.g., metal implants and claustrophobia).

Although TCD is inexpensive and its sensitivity and specificity for monitoring of CBF is excellent, it is typically technically limited to short recording durations as minor movements of carefully placed probes may interrupt data collection.^47^ Semi-automated (robotic) TCD has been developed to facilitate extended, uninterrupted recordings using automated correction algorithms and flow velocity sampling methods to maintain optimum probe positioning.^47^ Robotic TCD allows more consistent and reproducible assessment of TCD metrics simplifying what was previously a highly operator-dependent intervention. As this technology becomes more autonomous and more integrated with plethysmograph and processing software, it may allow non-specialists to carry out the analysis in any outpatient or inpatient setting.

### Limitations and strengths

This pilot methodology has its limitations. First, even with the robotic probe assistance, there is a considerable learning curve for the correct use of TCD. Further, a consistent application of a suitable compression of the carotid arteries for THRT assessment requires some practice. With current mathematical modeling, a reliable nMxa is calculated using 30 min of recording after artefact removal and signal correction, meaning a minimum of 35–40 min of recording is required. Like with other methods that require a longer time to acquire signal, this makes such a protocol unsuitable for a proportion of patients. One participant, as part of his PCS, suffered from hyperactivity, difficulty concentrating, and early irritability, and resultantly, found the long periods of concentration required for the study difficult. Exploration of other indices of CA that can be performed over a shorter period is important to improve the feasibility and compliance of these techniques in contemporaneous practice.

Although the THRT can be performed over a short period of time, anecdotally, it is difficult in participants with certain anatomical characteristics (elevated body mass index [BMI], short necks) and sometimes uncomfortable for awake participants. Although there were slight differences between median nMxa and THRT values between sub-groups, our sample size is not intended to draw any conclusions about the relationship between nMxa and PCS. However, this methodology can be scaled to a larger patient population. Based on a mean nMxa or Mx in healthy adults of 0.21 (SD: 0.16) and abnormal values of Mx considered as >0.4,^42,48^ it is estimated that at least 100 participants distributed evenly across study groups are required to detect a difference with 90% power at a significance level of 5%.

## Conclusion

This pilot study has demonstrated a reproducible assessment of PCS symptoms and non-invasive measures of CA in five controls and five patients with TBI in a real-world setting. By scaling this methodology into a larger study, we hope to test whether changes in CA are correlated with symptomatic PCS in patients with a history of TBI.

## Supplementary Material

Supplemental data

## Abbreviations Used

ABP: arterial blood pressure
ASL: arterial spin labeling
BMI: body mass index
CA: cerebral autoregulation
CANTAB: Cambridge Neuropsychological Test Automated Battery
CBF: cerebral blood flow
CBFV: cerebral blood flow velocity
CRx: cerebrovascular reactivity
etCO_2_: end-tidal CO_2_
GCS: Glasgow Coma Scale
GOS-E: Glasgow Outcome Scale Extended
ICP: intracranial pressure
MAP: mean arterial blood pressure
MCA: middle cerebral artery
MRI: magnetic resonance imaging
mTBI: mild traumatic brain injury
NIH: National Institutes of Health
NINDS: National Institute of Neurological Disorders and Stroke
PAL: paired associated learning
PCS: post-concussion syndrome
PCSS: Post-Concussion Symptom Scale
PPPD: persistent postural-perceptual dizziness
SD: standard deviation
SF-36: Short Form 36 Health Survey
sRoR: static cerebral autoregulation
TBI: traumatic brain injury
TCD: transcranial Doppler
THRR: transient hyperaemic response ratio
THRT: Transient Hyperaemic Response Test
VVAS: Vestibular Visual Analogue Scale
