# Supplementary material for: Robotic Semi-Automated Transcranial Doppler Assessment of Cerebrovascular Autoregulation in Post-Concussion Syndrome: Methodological Considerations
Source: Neurotrauma Rep. 2020 Nov 25;1(1):218–31. doi: 10.1089/neur.2020.0021 (PMC7703686; doi:10.1089/neur.2020.0021)
Supplement: Supplemental data [file Supp_FigS1.pdf]

Non-invasive measurement of cerebral blood flow regulation in post-concussive syndrome-like presentations: feasibility & correlations

Participant Demographics & Symptoms Questionnaire

Principal Investigator: Adel Helmy
Study Lead: Danyal Khan
IRAS: 266210

I confirm that I have read the relevant Participant Information Sheet (insert version number/date) and signed the Study Consent Form.
I have had the opportunity to consider the information and ask any questions I may have. I understand that my participation is voluntary and that I am free to withdraw at any time without giving any reason, without my medical care or legal rights being affected.

☐ Yes
☐ No

If no, please do not proceed through the rest of the questions below.

Name:
Prefix (circle appropriate) – Mr. /Mrs. /Ms. /Dr /Prof

First & Last Name:

Biological Sex (circle appropriate):

Female / Male / Prefer not to say / Other

If other, please specify:

Age:

Preferred email address (optional):

Other medical conditions
For example cardiovascular, respiratory, renal, neurological, ontological, psychiatric conditions.

Cardiovascular medications:
For example medications that lower your blood pressure, diuretic “water” table, medications that slow done your heart rate, etc.

Sports (circle appropriate):
Yes / No

If yes, does this include contact sports?:

Occupation:

Smoking history:
Current smoker (circle appropriate)?:
Yes / No

If yes, how many packs x how many years?:

If yes, any consumption within last 48 hours?:

If no, previous smoker? (circle appropriate):
Yes / No

If yes, how many packs x how many years?

Do you drink alcohol (circle appropriate)?:
Yes / No

If yes, units per week:

If yes, any consumption within last 48 hours? (circle appropriate):
Yes / No

Do you use any Illicit substances (circle appropriate)?:
Yes / No

If yes, please specify:

If yes, any consumption within last 48 hours? (circle appropriate):
Yes / No

Caffeine (e.g. coffee, tea, energy drinks)
Any last consumption if within last 48 hours? (circle appropriate):
Yes / No

If yes, how recently?:

Post-concussion syndrome symptoms:

Date of last concussion:

Please tick as appropriate in the table below:

Post-Concussion Symptom Scale (PCSS) Score - at rest							
Symptom	None	Mild		Moderate		Severe	
	0	1	2	3	4	5	6
Headache							
Nausea							
Vomiting							
Balance problems							
Dizziness							
Fatigue							
Trouble falling asleep							
Sleeping more than usual							
Sleeping less than usual							
Drowsiness							
Sensitivity to light							
Sensitivity to noise							
Irritability							
Sadness							
Nervousness							
Feeling more emotional							
Numbness or tingling							
Feeling slowed down							
Feeling mentally "foggy"							
Difficultly concentrating							
Difficulty remembering							
Total Score							

Duration of symptom (months/years):

Do the symptoms change on exertion (circle appropriate):
Yes / No

Management:

Traumatic Brain Injury:

Date of injury:

Severity: Were you told your head injury was mild, moderate or severe?

Did you have loss of consciousness at time of injury (circle appropriate):
Yes / No

Did you have amnesia after injury (circle appropriate):
Yes / No

Do you have any neurological deficits (weakness of muscles, numbness or tingling, problems with speech/language, etc DUE TO your head injury):

Management of your head injury (are you on any new medications for your head injury? Did you have any surgery for your head injury?):

Vestibular Profile:

Please tick as appropriate in the tables below:

Vestibular Visual Analogue Score -at rest											
Please indicate the amount of dizziness you experience in the following situations by marking off the scales below. 0 represents no dizziness and 10 represents the most dizziness											
Situation	0	1	2	3	4	5	6	7	8	9	10
Walking through a supermarket aisle											
Being a passenger in a car											
Being under fluorescent lights											
Watching traffic at a busy intersection											
Walking through a shopping mall											
Going down an escalator											
Watching a movie at the movie theatre											
Walking over a patterned floor											
Watching action television											

Vestibular Symptom Checklist		
	**Please tick if yes**	**Please tick if no**
**Most prominent symptoms:**		
Actual spinning		
Light-headedness		
Imbalance and unsteadiness		
“Brain-fog”		
Side to side swaying or front back swaying		
**Duration of dizziness episodes:**		
Seconds to minutes		
Minutes to hours		
Hours to days		
All of the time		
**Dizziness is brought on by:**		
Standing from sitting		
Rolling over in bed		
Stress		
Physical exertion		
When out in complicated visual environments (striped walls, shopping markets, chequered floors)		

How long have you suffered from these symptoms? (months):

Do you suffer from any inner ear diseases (for example vertigo, tinnitus, dizziness)? (circle appropriate):
Yes / No

Do you suffer from migraines? (circle appropriate):
Yes / No

Previous vestibular function tests (circle appropriate):
Yes / No

If yes, were they abnormal?

Previous abnormal imaging (circle appropriate):
Yes / No

Previous management (are you on any new medications for your inner ear disease? Did you have any surgery for your inner ear disease?):

Post study feedback:

How acceptable did you find the study process? (circle appropriate):

1 - Not at all acceptable
2 - Somewhat acceptable
3 - Acceptable
4 - Very acceptable
5 - Extremely acceptable

What did you enjoy about study participation?

What did you find difficult about the study process?

Do you have any other feedback for the study team?
